# Diagnostic Accuracy of the STANDARD F TB-Feron FIA Assay for Tuberculosis Infection in Vietnam: A Cross-Sectional Study

**DOI:** 10.1093/cid/ciaf561

**Published:** 2025-11-26

**Authors:** Han Thi Nguyen, Luan Nguyen Quang Vo, Andrew James Codlin, Rachel Forse, Tom Wingfield, Kristi Sidney Annerstedt, Emily Lai-Ho MacLean, Jacob Creswell, Beatrice Kirubi, Hoa Binh Nguyen, Luong Van Dinh, Ha Thu Doan, Lina Davies Forsman, for the TBI Testing Team, Dinh Van Luong, Nguyen Binh Hoa, Ha Thi Tuyet Trinh, Doan Thu Ha, Dinh Thi Huong, Nguyen Trung Thanh, Nguyen Thi Han, Nguyen Thi Cam Van, Ong Nguyen Huyen Trang, Tran Thi Thu Thuy, Luan Vo Nguyen Quang, Andrew James Codlin, Bui Thi Huyen, Lina Davies Forsman

**Affiliations:** Research Department, FIT RD Social Enterprise Company Limited (FIT RD), Hanoi, Vietnam; Division of Infectious Diseases, Department of Medicine, Karolinska Institutet, Stockholm, Sweden; Management, Friends for International TB Relief, Hanoi, Vietnam; Department of Global Public Health, Karolinska Institutet, Stockholm, Sweden; Management, Friends for International TB Relief, Hanoi, Vietnam; Department of Global Public Health, Karolinska Institutet, Stockholm, Sweden; Management, Friends for International TB Relief, Hanoi, Vietnam; Department of Global Public Health, Karolinska Institutet, Stockholm, Sweden; Department of Global Public Health, Karolinska Institutet, Stockholm, Sweden; Centre for Tuberculosis Research, Departments of Clinical Sciences and International Public Health, Liverpool School of Tropical Medicine, Liverpool, United Kingdom; Tropical and Infectious Diseases Unit, Liverpool University Hospitals NHS Foundation Trust, Liverpool, United Kingdom; Department of Global Public Health, Karolinska Institutet, Stockholm, Sweden; NHMRC Clinical Trials Centre, Faculty of Medicine and Health, The University of Sydney, Sydney, NSW, Australia; TB REACH, Stop TB Partnership, Geneva, Switzerland; TB REACH, Stop TB Partnership, Geneva, Switzerland; Research Centre, Vietnam National Lung Hospital, Hanoi, Vietnam; Research Centre, Vietnam National Lung Hospital, Hanoi, Vietnam; Research Centre, Vietnam National Lung Hospital, Hanoi, Vietnam; Division of Infectious Diseases, Department of Medicine, Karolinska Institutet, Stockholm, Sweden; Department of Infectious Diseases, Karolinska University Hospital, Stockholm, Sweden

**Keywords:** TB, QuantiFERON-TB Gold Plus, point of care, fluorescent immunoassay, interferon-gamma release assays

## Abstract

**Background:**

Tuberculosis (TB) infection is a driver of the global TB epidemic. Accurate, affordable, and simpler diagnostics are crucial for identifying people for preventive therapy. We evaluated the diagnostic performance of the STANDARD F TB-Feron FIA (TB-Feron), a near-point-of-care (POC) assay for detecting TB infection.

**Methods:**

From June to December 2024, we conducted a cross-sectional study at the Vietnam National Lung Hospital, enrolling 352 participants, including 345 eligible participants: 95 with microbiologically confirmed pulmonary TB (Group 1), 200 household contacts of people with pulmonary TB (Group 2), and 50 with a recent history of a negative QFT-Plus result and no known TB exposure (Group 3). Participants were tested with TB-Feron and the reference standard, QuantiFERON TB Gold Plus (QFT-Plus). Results were compared for sensitivity and specificity (primary endpoints), with inter-test agreement (Cohen's κ) and reproducibility (Bland–Altman analysis) as secondary outcomes.

**Results:**

Among 345 eligible participants, TB-Feron sensitivity was 88.4% (95% confidence interval [CI] 80.2–94.1) in Group 1, and specificity was 70.0% (55.4–82.1) in Group 3. In Group 2, positive and negative agreements with QFT-Plus were 89.2% (79.8–95.2%) and 75.4% (66.9–82.6), respectively, with inter-test agreement of 80.5% (Cohen's κ=0.6069, *P* < .0001). Intra-test reproducibility showed no significant differences in IFN-γ levels (mean difference = 2.08 IU/mL, 95% CI −1.28 to 5.44, *P* = .206).

**Conclusions:**

With high sensitivity, the TB-Feron assay is a potential near-POC alternative to the QFT-Plus assay for diagnosing TB infection, but requires consideration of its suboptimal specificity.

## Teaser

The study evaluated the diagnostic performance of the near-point-of-care TB-Feron assay in Vietnam, demonstrating high sensitivity and good reproducibility, which supports its potential use for TB infection screening in high-burden, resource-limited settings, despite its moderate specificity.

## Graphical Abstract

**Graphical Abstract:**
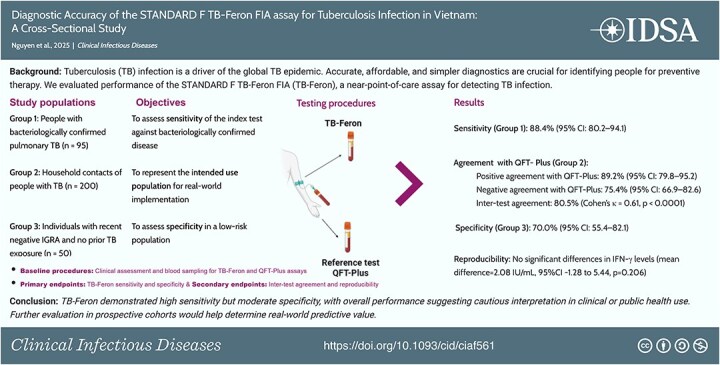
https://tidbitapp.io/institutional-portal/clinical-infectious-diseases/tidbits/diagnostic-accuracy-of-the-standard-f-tb-feron-fia-assay-for-tuberculosis-infection-in-vietnam-a-cross-sectional-study-f8f63d29-a6a3-466c-8288-a2e321d0da60/update

Tuberculosis (TB) remains a global health crisis, causing 1.25 million deaths in 2023. In Vietnam, 1 of the 30 highest TB burden countries, 182 000 people newly developed TB in 2023, driven by progression of infection to disease [1]. Globally, 1.7–2.3 billion people may harbor infection, with 5–10% progressing to active TB within 2 years [2,3]. Accurate diagnosis of TB infection is crucial for identifying individuals who could benefit from preventive therapy and achieving WHO End TB targets [1,4].

Current diagnostic tools, including the tuberculin skin test (TST) and interferon-gamma release assays (IGRAs), are limited by suboptimal sensitivity, particularly among immunocompromised individuals [5,6]. TSTs suffer from low specificity in Bacillus Calmette–Guérin (BCG)-vaccinated populations and require follow-up visits, leading to loss to follow-up [7,8]. IGRAs, such as the QuantiFERON-TB Gold Plus assay (QFT-Plus; QIAGEN, Venlo, Netherlands), improve specificity by detecting IFN-γ responses to TB-specific antigens (ESAT-6 and CFP-10), but require advanced laboratory infrastructure and skilled personnel, restricting use in resource-constrained settings [9]. There is a growing need for novel, point-of-care (POC) tests that are affordable, operationalizable at the primary care level and capable of streamlining workflows while minimizing costs [10].

The STANDARD F TB-Feron FIA assay (TB-Feron; SD BIOSENSOR, Gyeonggi-do, South Korea) is a simple fluorescent immunoassay and IGRA measuring IFN-γ responses to ESAT-6, CFP-10, and TB 7.7 antigens. Using a single TB antigen tube alongside nil and mitogen tubes, it provides results 15 min post-incubation via a portable analyzer, facilitating rapid TB infection screening in resource-limited settings [11]. Published evidence on its accuracy is limited, and no direct comparisons to established IGRAs in high TB burden countries.

We hypothesized that the TB-Feron offers comparable diagnostic accuracy to QFT-Plus, making it a feasible near-POC test for TB infection detection in Vietnam. We aimed to evaluate its performance against QFT-Plus, specifically assessing sensitivity in adults with confirmed pulmonary TB and its accuracy across diverse risk groups.

## METHODS

### Study Design and Participants

This prospective, blinded, cross-sectional diagnostic accuracy study was conducted at the Vietnam National Lung Hospital (NLH), Hanoi, between June and December 2024. Ethical approval was obtained from the NLH Institutional Review Board (#65/23/CN-HDDD-BVPTU) and the Swedish Ethical Review Authority (#2023-04271-01), following the protocol [12]. The trial was registered at ClinicalTrials.gov (NCT06221735).

Participants were recruited into 3 groups from the NLH outpatient clinic, a national tertiary referral center. Group 1 comprised adults (aged ≥18 years) with microbiologically confirmed pulmonary TB by Xpert MTB/RIF Ultra assay (Cepheid, United States) and abnormal chest X-rays (CXR). Group 2 included asymptomatic household contacts (HHCs) of individuals with pulmonary TB who had normal CXR. HHCs were defined as individuals sharing a residence or kitchen with the index case ≥ one night/week for 3 months preceding the diagnosis, or ≥1 h/day for 5 days/week [4]; multiple HHCs per index person were allowed. Group 3 consisted of individuals with a prior negative QFT-Plus result (within six months), no known TB exposure, and normal CXR.

This grouping approach and analytic framework were based on the WHO consolidated guidelines on TB infection diagnostics [13–15]: Group 1 assessed sensitivity, Group 3 assessed specificity, and Group 2 represented the intended use population for real-world implementation. Exclusion criteria were presumptive TB in Groups 2 and 3 and prior TB diagnosis or treatment. All participants gave written informed consent.

### Procedures

Venous blood (10 mL) was collected for simultaneous TB-Feron and QFT-Plus testing. Group 1 participants were recruited after microbiological confirmation of pulmonary TB by Xpert MTB/RIF Ultra and abnormal CXR, but before treatment. Xpert Ultra “trace” results underwent repeat Xpert Ultra and liquid culture for confirmation. Groups 2 and 3 were enrolled via a dedicated study pathway outside routine services.

Samples were drawn into QFT-Plus tubes (nil, TB1, TB2, and mitogen) and TB-Feron tubes (nil, TB antigen, and mitogen) and transported fresh to the Vietnam National Reference Laboratory (VNRL), within 2 h. TB-Feron tubes were incubated at 37°C for 16–24 h and analyzed using the STANDARD F2400 fluorescence immunoassay [11]. QFT-Plus tubes were processed per manufacturer's instructions using ELISA [16]. Positivity for both assays was determined using manufacturer-defined cutoffs (≥0.35 IU/mL). Laboratory personnel performing the TB-Feron were blinded to clinical data and QFT-Plus results.

To assess intra-test reproducibility, 15 participants were randomly selected to provide duplicate blood samples, analyzed independently by 2 technicians at VNRL.

### Outcomes

The primary outcomes were TB-Feron sensitivity and specificity, defined per WHO consolidated guidelines on TB infection diagnostics [13, 14]. Secondary outcomes included inter-test agreement with QFT-Plus (Cohen's κ) and intra-test reproducibility based on duplicate testing. Distributions of IFN-γ levels were evaluated across study groups.

### Statistical Analysis

Sample sizes were based on prior TB-Feron estimates (sensitivity 88.9%, specificity 92.5%) [17], with 10% dropout, requiring minimum of 38 (Group 1), 85 (Group 2), and 29 (Group 3). To improve precision and enable subgroup analyses, we over-recruited to 95, 200, and 50 participants, respectively, anticipating up to 10% indeterminate results [18].

Sensitivity and specificity were estimated following WHO recommendations [13, 15]. In Group 2, positive and negative agreement with QFT-Plus, positive predictive value (PPV), negative predictive value (NPV), and 95% confidence intervals (CIs) were calculated. As multiple contacts could be recruited per index (mean 2.08), the main analysis (n = 200) did not adjust for clustering due to missing index identifiers for 23 contacts. A sensitivity analysis was conducted in the 177 contacts with identifiers using generalized estimating equations (GEEs) with an independent correlation structure [19].

Area under the curve (AUC) was reported. Cohen's κ [20] measured inter-test agreement. Bland–Altman plots evaluated intra-test reproducibility, calculating difference and coefficient of repeatability [21].

Multivariable logistic regression explored predictors of TB-Feron and QFT-Plus discordance, with study group as main exposure and covariates (age, sex, BCG status, smoking, TB exposure) selected a priori. Complete-case analyses were performed.

Post hoc analyses assessed IFN-γ patterns in concordant vs discordant results and the impact of coinfections. While both assays report IFN-γ in IU/mL, values are not directly comparable due to differing antigens and platforms; comparisons were used descriptively to explore response trends.

Significance was set at *P* < .05, using Stata version 15 (StataCorp, College Station, TX, United States).

## RESULTS

### Clinical Characteristics

**Figure 1.**
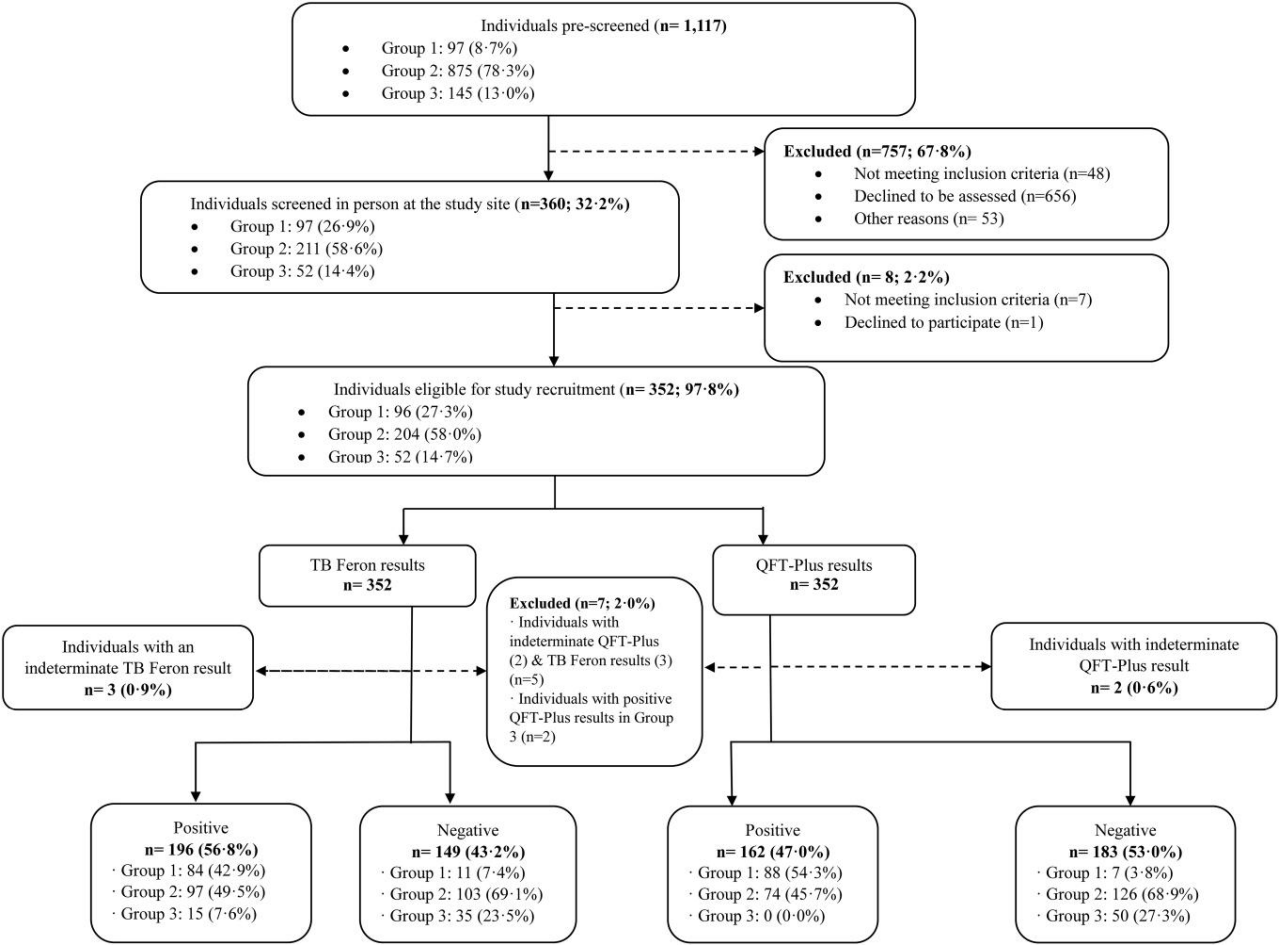
Flow diagram of participant enrollment, exclusion, and final analysis. Between June and December 2024, 1117 individuals were screened, 352 were enrolled, and 345 were included in the final analysis (95 microbiologically confirmed tuberculosis (TB), 200 household contacts, and 50 negative controls). Alt text: Flow chart depicting participant screening, enrollment, exclusions, and final analysis groups.

Between June and December 2024, 1117 participants were screened. Of these, 97 (8.7%) were categorized into Group 1 (microbiologically confirmed TB), 875 (78.3%) into Group 2 (HHCs), and 145 (13.0%) into Group 3 (negative control). We excluded 757 (67.8%) participants: 48 (4.3%) ineligible, 656 (58.7%) declined, and 53 (4.7%) for other reasons. Subsequently, 7 individuals were excluded for ineligibility, and one declined participation. Of 352 participants enrolled, 345 were included in the final analysis after excluding 5 indeterminate results (3 TB-Feron [0.9%] and 2 QFT-Plus [0.6%]) and 2 Group 3 participants with QFT-Plus seroconversion. The final cohort comprised 95 in Group 1, 200 in Group 2, and 50 in Group 3 (Figure 1; Supplementary Table 5).

The median age was 40 years (IQR 28–51), with 157 (45.5%) male participants. Smoking was reported by 75 (21.7%) participants and alcohol use by 107 (31.0%), of whom 99 (28.7%) consumed 1–7 units per week [22]. Diabetes mellitus was reported in 10 (2.9%) participants, and 137 (39.7%) had no BCG vaccination. Twelve (3.5%) reported other current infections, including influenza, dental caries, pharyngitis, keratitis, and postoperative infection following breast surgery, pneumonia, and otitis externa. In Group 1, 88 (92.6%) reported cough, 11 (11.6%) hemoptysis, 20 (21.1%) night sweats, and 2 (2.1%) fever. By TB symptoms score, 82 (86.3%) had mild symptoms [23], and 13 (13.7%) had moderate symptoms. QFT-Plus positivity was higher among men (53.1%) than women (46.9%) (Table 1; Supplementary Tables 3a and 3b). Although HIV or other forms of immunosuppression were not exclusion criteria, none were enrolled.

**Table 1.** Baseline Characteristics of Study Participants. QuantiFERON Gold-Plus TB Test results

Characteristics	Group 1 (n = 95)				*P* Value	Group 2 (n = 200)				*P* Value	Group 3 (n = 50)			
	Negative		Positive			Negative		Positive			Negative		Positive	
	n	%	n	%		n	%	n	%		n	%	n	%
Total														
Sex	7	7.37%	88	92.6%	.062	126	63.0%	74	37.0%	.678	50	100.0%	0	0.0%
Male	7	100.0%	58	65.9%	…	44	34.9%	28	37.8%	…	20	40.0%	…	…
Female	0	0.0%	30	34.1%	…	82	65.1%	46	62.2%	…	30	60.0%	…	…
Age group (years)					.732					.048				
18–34	3	42.9%	32	36.4%	…	40	31.7%	15	20.2%	…	50	100.0%	…	…
35–44	0	0.0%	15	17.1%	…	31	24.6%	25	33.8%	…	…	0.0%	…	…
45–54	1	14.3%	17	19.3%	…	37	29.4%	15	20.3%	…	…	0.0%	…	…
55–64	2	28.6%	14	15.9%	…	15	11.9%	13	17.6%	…	…	0.0%	…	…
>64	1	14.2%	10	11.3%	…	3	2.4%	6	8.1%	…	…	0.0%	…	…
Median age (IQR)^c^	51	30–63	42	30–57	.7647	43.5	32–50	43	35–55	.1834	25	22–26	…	…
Smoking^a^	3	42.9%	34	38.6%	.826	14	11.1%	19	25.7%	.007	5	10.0%	…	…
Alcohol	…	0.0%	…	0.0%	…	…	0.0%	…	0.0%	…	…	0.0%	…	…
No alcohol	5	71.4%	53	60.2%	0.039	89	70.6%	53	71.6%	.192	38	76.0%	…	…
1–7 units/week	1	14.3%	34	38.6%	…	36	28.6%	17	23.0%	…	11	22.0%	…	…
8–14 units/week	1	14.3%	1	1.1%	…	1	0.8%	3	4.0%	…	1	2.0%	…	…
>14 units/week	0	0.0%	0	0.0%	…	0	0.00%	1	1.4%	…	0	0.0%	…	…
Diabetes mellitus type II	1	14.3%	7	8.0%	.562	2	1.6%	0	0.0%	.276	0	0.0%	…	…
HBV/HBC coinfection	0	0.0%	2	2.3%	.687	5	4.0%	3	4.1%	.976	0	0.0%	50	…
BCG vaccinated					.74					.344				
No vaccination	4	57.1%	47	53.4%	…	46	36.5%	34	46.0%	…	6	12.0%	…	…
Vaccinated	3	42.9%	34	38.6%	…	37	29.5%	16	21.6%	…	37	74.0%	…	…
Unknown	0	0.0%	7	8.0%	…	43	34.1%	24	32.4%	…	7	14.0%	…	…
TB symptoms	7	100.0%	82	93.2%	.475	…	0.0%	…	0.0%	…	…	0.0%	…	…
Cough	7	100.0%	81	92.1%	.438	…	0.0%	…	0.0%	…	…	0.0%	…	…
Hemoptysis	1	14.3%	10	11.4%	.816	…	0.0%	…	0.0%	…	…	0.0%	…	…
Night sweats	2	28.6%	18	20.5%	.636	…	0.0%	…	0.0%	…	…	0.0%	…	…
Fever	0	0.00%	2	2.3%	.687	1	0.8%	0	0.0%	.442	0	0.0%	…	…
TB score^b^ [20]					.962									
Mild	6	85.7%	76	86.4%	…	…	0.0%	…	0.0%	…	…	0.0%	…	…
Moderate to severe	1	14.3%	12	13.6%	…	…	0.0%	…	0.0%	…	…	0.0%	…	…
Other infections	0	0.0%	0	0.0%	NA	2	1.6%	6	8.1%	.023	4	8.0%	…	…

QuantiFERON Gold-Plus TB test results. χ² tests were used for all other values. Group 1: microbiologically confirmed pulmonary TB; Group 2: asymptomatic household contacts; Group 3: negative control.

Abbreviations: HBC, hepatitis C virus; HBV, hepatitis B virus; IQR, interquartile range; NA, not applicable; TB, tuberculosis.

^a^Smoking: including current and past smoking.

^b^TB score: 0–5 = mild, 6–11 = moderate to severe.

^c^Mann–Whitney *U*-test (skew distribution).

### Diagnostic Performance

Among 345 participants, TB-Feron was positive in 196 (56.8%) and QFT-Plus in 162 (47.0%) (Supplementary Tables 3b and 4).

In Group 1 (microbiologically confirmed pulmonary TB), TB-Feron sensitivity was 88.4% (84/95; 95% CI 80.2–94.1) when evaluated against the WHO-defined benchmark of microbiologically confirmed disease. In Group 3 (low-risk controls), TB-Feron specificity was 70.0% (35/50; 95% CI 55.4–82.1), based on WHO criteria for evaluating specificity among individuals with no known TB exposure, normal CXR, and prior negative QFT-Plus results (Table 3a) [13, 14].

In Group 2 (HHCs), TB-Feron was positive in 97 of 200 participants (48.5%), and QFT-Plus was positive in 74 of 200 (37.0%) (Table 2). Using QFT-Plus as a reference, positive and negative agreements between TB-Feron and QFT-Plus were 89.2% (95% CI 79.8–95.2) and 75.4% (95% CI 66.9–82.6), respectively. The AUC for TB-Feron was 0.82 (95% CI 0.77–0.87), based on an IFN-γ positivity threshold of ≥0.35 IU/mL. The PPV was 68.0% (95% CI 57.8–77.1), and the NPV was 92.2% (95% CI 85.3–96.6). Overall agreement between the 2 assays was 80.5% (Cohen's κ=0.607, *P* < .001). In the sensitivity analysis restricted to 177 contacts with available index identifiers, GEE-adjusted estimates were 89.4% (95% CI, 82.2–96.5) for positive agreement, 73.9% (95% CI, 65.4–82.3) for negative agreement, 67.0% (56.8–77.3%) for PPV, and 92.1% (86.5–97.8%) for NPV, consistent with the unadjusted results (Table 3b).

**Table 2.** Cross-Tabulation of TB-Feron and QFT-Plus Test Results

		Group 1 (95)		*P* Value		Group 2 (200)		*P* Value		Group 3 (50)		*P* Value
		QFT-Plus				QFT-Plus				QFT-Plus		
		Positive 88 (92.6%)	Negative 7 (7.4%)			Positive 74 (37.0%)	Negative 126 (63.0%)			Positive 0 (0.0%)	Negative 50 (100.0%)	
TB-Feron	Positive 84 (88.4%)	80 (95.2%)	4 (4.8%)	.007	Positive 97 (48.5%)	66 (68.0%)	31 (32.0%)	<.0001	Positive 15 (30.0%)	0 (0.0%)	15 (30.0%)	NA
	Negative 11 (11.6%)	8 (72.7%)	3 (27.3)		Negative 103 (51.5%)	8 (7.8%)	95 (92.2%)		Negative 35 (70.0%)	0 (0.0%)	35 (70.0%)	

Group 1: microbiologically confirmed pulmonary TB; Group 2: asymptomatic household contacts; Group 3: negative control.

Abbreviations: NA, not applicable; TB, tuberculosis.

**Table 3a.** Sensitivity and Specificity of TB-Feron

	Sensitivity (95% CI)	Specificity (95% CI)
Group 1 (n = 95)	88.4% (80.2–94.1%)	Not applicable
Group 3 (n = 50)	Not applicable	70.0% (55.4–82.1%)

**Table 3b.** Positive and Negative Agreement, Predictive Values, and AUC of TB-Feron Compared With QFT-Plus in Group 2 (Household Contacts)

	Positive Agreement (95% CI)	Negative Agreement (95% CI)	Positive Predictive Value (95% CI)	Negative Predictive Value (95% CI)	ROC Area (AUC) (95% CI)	Agreement	Cohen's Kappa (k)	*P* Value
Group 2 (n = 200)	89.2% (79.8–95.2%)	75.4% (66.9–82.6%)	68.0% (57.8–77.1%)	92.2% (85.3–96.6%)	0.82 (0.77–0.87)	80.5%	0.6069	<.00001
Group 2* (n = 177)	89.4% (82.2–96.5%)	73.9% (65.4–82.3%)	67.0% (56.8–77.3%)	92.1% (86.5–97.8%)	0.82 (0.76–0.87)	NA	NA	NA

Group 2: all 200 household contacts, unadjusted for clustering. Group 2*: subset of 177 contacts with index identifiers: sensitivity, specificity, NPV, PPV, and AUC estimated using generalized estimating equations (GEE) with independent correlation to account for clustering by index case. Other metrics (agreement, κ) were reported unadjusted, as GEE-based estimation methods are not available.

Abbreviations: PPV, positive predictive value; NPV, negative predictive value; ROC, receiver operating characteristic; AUC, area under the curve; CI, confidence intervals; NA, not applicable; TB, tuberculosis.

### Factors Associated With Discordance Between TB-Feron and QFT-Plus

Multivariable logistic regression in full cohorts (n = 345) assessed factors associated with discordant TB-Feron and QFT-Plus results, adjusting for group. Discordance was defined as a qualitative mismatch between the 2 test outcomes. In adjusted analysis, the presence of cough was associated with lower odds of discordance (adjusted odds ratio [aOR] 0.46, 95% CI .22–.94; *P* = .034). Participants reporting concurrent non-TB infections, such as influenza, pneumonia, or pharyngitis, had higher odds of discordance (aOR 4.55, 95% CI 1.42–14.60; *P* = .011). No significant associations were observed for age, sex, BCG vaccination status, diabetes, or smoking history. Full model covariates and estimates are reported in Table 4 and Supplementary Table 6a and b.

**Table 4.** Univariable and Multivariable Logistic Regression Analysis of Participant Covariates Associated With Discordance Between TB-Feron and QFT-Plus Results

	aOR	*P*	95% CI	OR	*P*	95% CI
Gender (male vs female)	0.69	.174	.40; 1.18	0.37	.01	.17; .79
Age group (years)						
18–34	Ref.		…	Ref.	…	…
35–44	0.79	.518	.38; 1.62	0.72	.49	.28; 1,82
45–54	0.66	.274	.31; 1.40	0.75	.566	.28; 2,01
55–64	0.67	.375	.27; 1.64	0.61	.396	.19; 1.91
>65	0.88	.828	.27; 2.82	1.27	.739	.31; 5.24
Smoking (yes vs no)	0.76	.437	.38; 1.51	0.54	.182	.22; 1.33
Diabetes (yes vs no)	0.46	.467	.06; 3.71	0.74	.793	.08; 6.98
HBC/HCV coinfection (yes vs no)	2.94	.103	.80; 10.71	4.8	.031	1.16; 19.90
BCG vaccination						
Yes	1.21	.542	.65; 2.27	1.06	.896	.44; 2.57
No	Ref.		…	Ref.	…	…
Unknown	1.4	.322	.71; 2.82	1.39	.396	.65; 2.95
Alcohol use						
None	Ref.		…	Ref.	…	…
1–7 units	0.95	.876	.52; 1.75	0.69	.371	.31; 1.55
8–14 units	4.29	.081	.84; 21.95	4.54	.128	.65; 31.79
>14 units	Ref.		…	Ref.	…	…
TB symptoms (yes vs no)	0.45	.031	.22;.93	1.46	.988	…
Cough (yes vs no)	0.46	.034	.22;.94	Ref.	…	…
Hemoptysis (yes vs no)	0.93	.928	.19; 4.41	0.29	.321	.03; 3.35
Fever (yes vs no)	Ref.		…	Ref.	…	…
Night sweat (yes vs no)	1.05	.935	.34; 3.24	4.06	.089	.81; 20.37
Disease severity by TB score^a^						
Mild	Ref.		…	Ref.	…	…
Moderate to severe	1.94	.285	.58; 6.49	9.64	.021	1.40; 66.48
Other infections	0.22	<.0001	.17;.29	4.55	.011	1.42; 14.60

Abbreviations: HBC, hepatitis C virus; HBV, hepatitis B virus; TB, tuberculosis.

^a^TB score: 0–5 = mild, 6–11 = moderate to severe.

### Interferon-Gamma Levels and TB-Feron Results

Post hoc analysis of IFN-γ concentrations was performed in the full study population (n = 345) to explore patterns underlying concordant and discordant TB-Feron and QFT-Plus results. The mean corrected IFN-γ level (TB2-nil) was 2.14 IU/mL (95% CI 1.80–2.49). Among TB-Feron-positive individuals, the mean IFN-γ concentration was 3.56 IU/mL (95% CI 3.05–4.06), compared with 0.28 IU/mL (95% CI .06–.50) among those testing negative. For QFT-Plus, the corresponding values were 4.52 IU/mL (95% CI 4.00–5.04) for positives and 0.04 IU/mL (95% CI −.08 to .15) for negatives. Among discordant TB-Feron results, false-positive cases had a mean IFN-γ level of −0.07 IU/mL (95% CI −.17 to .04), and false-negative cases had a mean of 2.00 IU/mL (95% CI .49–3.51); only 1 of the 8 false-negative cases occurred in a participant with a reported concurrent non-TB infection (Supplementary Tables 6b and 7a).

In Group 2 (HHCs), the mean corrected IFN-γ level was 1.30 IU/mL (95% CI .95–1.64). TB-Feron-positive individuals had a mean concentration of 2.50 IU/mL (95% CI 1.90–3.10), while TB-Feron-negative individuals had a mean of 0.17 IU/mL (95% CI −.03 to .36). For QFT-Plus, the corresponding values were 3.33 IU/mL (95% CI 2.65–4.01) for positives and 0.10 IU/mL (95% CI −.05 to .25) for negatives. False-positive TB-Feron results had a mean IFN-γ level of 0.01 IU/mL (95% CI −.06 to .07), while false-negative results had a mean of 0.56 IU/mL (95% CI .37−.75). Using the ≥0.35 IU/mL threshold, TB-Feron demonstrated a theoretical sensitivity of 74% and specificity of 91% in the full cohort, and 69% and 91%, respectively, in Group 2 (Supplementary Table 7b).

### Intra-test Reproducibility of TB-Feron

To evaluate the intra-test reproducibility of TB-Feron, 15 participants each provided 2 blood specimens, which were tested independently by 2 different laboratory staff using the TB-Feron assay. All paired tests yielded identical qualitative results (positive or negative) between staff. Bland–Altman analysis of IFN-γ levels showed a mean difference of 2.1 IU/mL (95% CI −1.28 to 5.44, *P* = .206), indicating no significant systematic bias between paired measurements. The 95% limits of agreement ranged from −9.8 IU/mL (95% CI −15.71 to −3.94) to 14.0 IU/mL (95% CI 8.10–19.87). The coefficient of repeatability was 12.2 IU/mL (95% CI 9.02–18.89) (Supplementary Figure 1 and Table 8).

## DISCUSSION

Our study found that TB-Feron performs well in detecting TB infection when evaluated using WHO-recommended case definitions [13, 14], with high sensitivity among individuals with microbiologically confirmed TB (88.4%, 95% CI 80.2–94.1) and moderate specificity in low-risk controls (70.0%, 95% CI 55.4–82.1). Among HHCs, the intended programmatic target population, TB-Feron demonstrated substantial agreement with QFT-Plus (80.5%, Cohen's κ=0.6069, *P* < .001). In a sensitivity analysis using GEE to adjust for clustering (177 contacts), estimates of positive (89.4%) and negative agreement (73.9%) were consistent with the unadjusted results, supporting our findings and the assay's potential programmatic use. These findings position TB-Feron as a promising near-POC assay for TB infection testing and may help address logistical barriers in resource-limited settings [24]. We also observed higher QFT-Plus positivity in men, consistent with global male predominance in TB infection and disease, including Vietnam [1, 25].

Our findings align with previous reports. TB-Feron sensitivity (88.4% in Group 1) and positive agreement with QFT-Plus (89.2% in Group 2) were comparable to results by Saint-Pierre et al, who reported 88.9% sensitivity against TST in a South American [17].

In Group 1 (microbiologically confirmed TB), QFT-Plus had a sensitivity of 92.6%, with 7 false-negative results when using the Xpert MTB/RIF Ultra assay as the reference standard. These false negatives may reflect limitations of IGRAs in detecting active TB, particularly in cases of extensive disease, as previously reported [26]. A recent meta-analysis reported a pooled QFT-Plus sensitivity of 94% (TB1 91%, TB2 95%) for active TB [27], highlighting its limitations and the continued absence of a gold standard for TB infection diagnosis due to false-negative results. However, TB-Feron's moderate specificity (70.0% in Group 3) and positive agreement with QFT-Plus (75.4% in Group 2) were lower than the 92.5% reported against TST, likely due to QFT-Plus being a more stringent reference standard that minimizes false positives in the reference standard among BCG-vaccinated populations [7, 28].

A recent study in Eastern Europe, a low drug-susceptible TB burden setting, evaluated the diagnostic accuracy of TB-Feron among 218 participants, including 103 with active TB, 68 with past TB, and 47 healthy controls [18]. Indeterminate results were more frequent than in our study, particularly for TB-Feron tests (9.2%) compared with QFT-Plus (2.3%), whereas in our high-burden setting, indeterminate rates were below 1% for both assays (0.9% for TB-Feron and 0.6% for QFT-Plus). In that study, TB-Feron FIA sensitivity in active and past TB cohorts was 82–85%, and specificity in healthy controls was 87% [18]. Compared with our findings in Vietnam, these results indicate that TB-Feron achieves higher sensitivity but lower specificity in high-burden settings, likely due to increased TB infection prevalence and non-tuberculous mycobacteria (NTM) exposure. A meta-analysis reported QFT-Plus specificities above 96% in healthy individuals, but lower specificity in HIV-positive cohort (82.6%), suggesting TB-Feron's moderate specificity in Group 3 may reflect immune suppression or cross-reactivity with NTM [6, 27].

TB-Feron's high NPV (89.3% overall, 92.2% in Group 2) supports its utility in ruling out TB infection, particularly among HHCs. This aligns with findings from Rangaka et al that IGRAs with high NPV can effectively guide preventive therapy in high-risk groups [29]. However, the moderate specificity in Group 3 raises concerns about false positives, potentially leading to unnecessary treatment and resource use [30]. WHO also advises against using IGRA to diagnose active TB, as positive results may reflect prior infection or alternative conditions [13, 14].

The higher IFN-γ levels in TB-Feron-positive (3.56 IU/mL) and QFT-Plus-positive (4.52 IU/mL) results compared with negative results (0.28 IU/mL and 0.04 IU/mL, respectively) indicate robust antigenic stimulation, consistent with ESAT-6 and CFP-10 responses in infected individuals. Slightly higher IFN-γ in TB-Feron-negative individuals compared with QFT-plus negative likely reflects differences in assay design, including the antigens used, the contribution of both CD4 and CD8 T cells, and nil-correction methods [11, 16] (Supplementary Tables 1 and 2). These differences are within the expected technical and biological variability and do not alter the classification of negative results [31]. The mean IFN-γ level in false-negative TB-Feron results (2.00 IU/mL) suggests potential limitations in the ≥0.35 IU/mL threshold, particularly in Group 1 (microbiologically confirmed TB), where immune suppression may reduce IFN-γ responses, as reported in advanced TB cases [27]. This contrasts with QIAreach, which uses a lower threshold (≥0.20 IU/mL), which may improve sensitivity, but risks lower specificity [32, 33].

TB-Feron showed substantial inter-test agreement with QFT-Plus among HHCs (Cohen's κ=0.6069) and robust intra-test reproducibility (mean difference 2.08 IU/mL, *P* = .206; repeatability 12.20 IU/mL). The Bland–Altman analysis confirmed no significant bias in IFN-γ measurements between laboratory staff, supporting its feasibility in settings with limited infrastructure and decentralized use. Compared with QIAreach, which also demonstrates high reproducibility (coefficient of variation < 10%), TB-Feron's broader limits of agreement (−9.83 to 13.99 IU/mL) suggest greater variability in IFN-γ quantification, potentially due to fluorescence-based detection vs QIAreach's digital reader [33].

Logistic regression showed that cough and the presence of any TB symptom reduced discordance, suggesting symptoms enhance diagnostic concordance in active TB, aligning with ATS/IDSA guidelines [34]. In contrast, concurrent infections increased discordance, consistent with evidence that acute infections modulate immune response and interfere with INF-γ release. A previous study also reported indeterminate or discordant IGRA results during viral infections and systemic inflammation [35]. Hepatitis B or C coinfection showed a trend toward increased discordance (aOR 2.94, *P* = .103), possibly due to immune dysregulation, as reported in a TB-HCV coinfection study [36]. No association with BCG vaccination reinforces IGRA's advantage over TST in Vietnam, where BCG coverage is high.

Strengths of this study include its prospective, blinded design, diverse cohorts, and multiple performance analyses. Limitations include the single-center design and lack of longitudinal follow-up, restricting generalizability and predictive insights. QFT-Plus's imperfect sensitivity may underestimate TB-Feron's accuracy. A further limitation is that multiple HHCs were recruited per index case, potentially introducing clustering. While the risk of clustering is expected to be very low (intraclass correlation coefficient = 0.008, as described in the Supplementary Appendix of Fox et al) [37], we nonetheless performed a conservative sensitivity analysis using GEE. Because identifiers were missing for 23 contacts, this adjustment was restricted to the 177 contacts with available identifiers.

## CONCLUSION

TB-Feron showed high sensitivity and reproducibility, supporting its use as a feasible alternative to QFT-Plus for TB infection screening in high-burden settings. Its near-POC format and simplicity may improve access to preventive therapy where conventional IGRAs are limited. However, moderate specificity may require confirmatory testing in low-prevalence populations. Further prospective validation in decentralized settings, including children and immunocompromised individuals, is warranted. Cost-effectiveness and longitudinal studies are needed to guide program integration and clarify its role in TB preventive treatment.

## Supplementary Material

ciaf561_Supplementary_Data

## Supplementary Data

Supplementary materials are available at *Clinical Infectious Diseases* online. Consisting of data provided by the authors to benefit the reader, the posted materials are not copyedited and are the sole responsibility of the authors, so questions or comments should be addressed to the corresponding author.
