# Supplementary material for: Diagnostic Accuracy of the STANDARD F TB-Feron FIA Assay for Tuberculosis Infection in Vietnam: A Cross-Sectional Study
Source: Clin Infect Dis. 2025 Nov 26;82(5):e996–e1004. doi: 10.1093/cid/ciaf561 (PMC13189665; doi:10.1093/cid/ciaf561)
Supplement: ciaf561_Supplementary_Data [file ciaf561_supplementary_data.zip › Supplementary Appendix_revised_19 Sept_clean.docx]

**Supplementary Appendix**

**Supplement to:**

Han Nguyen, Luan Vo, Andrew Coldin et al. Accuracy of the STANDARD F TB-Feron FIA assay for Tuberculosis Infection in Vietnam: A Cross-Sectional Study.

| **Table of Contents** | | **pages** |
| --- | --- | --- |
| 1 | List of members of the TBI Testing team | 3 |
| 2 | List of Ethics Boards and Regulatory Bodies | 4 |
| 3 | List of Inclusion and Exclusion Criteria | 5-6 |
| 4 | Supplementary methods, Tables S1-S2 | 7-8 |
| 5 | Supplementary Table S3a. Baseline characteristics of study participants (All populations by QFT-Plus) | 9-10 |
| 6 | Supplementary Table S3b. Baseline characteristics of study participants (by TB Feron results) | 11-12 |
| 7 | Supplementary Table S4. Cross-tabulation of TB-Feron and QFT-Plus test results (All populations) | 13 |
| 8 | Supplementary Table S5. TB Feron test results (including indeterminate results) | 14 |
| 9 | Supplementary Table S6a. Distribution of discordant IGRA results by study group and selected clinical factors | 15 |
| 10 | Supplementary Table S6b. Individual-level listing of discordant IGRA results with corresponding clinical factors | 16-18 |
| 11 | Table S7a. Relationship between IFN-γ level (TB2) and TB Feron results (All populations) | 19 |
| 12 | Table S7b. Relationship between IFN-γ level (TB2) and TB Feron results (Group 2- Household contacts) | 20 |
| 13 | Supplementary Table S8. Intra-Test Reproducibility of TB Feron | 21 |
| 14 | Supplementary Figure 1. Intra-Test Reproducibility of TB Feron (Bland Altman plot) | 22 |

**1. List of members of the TBI Testing team**

**Vietnam National Lung Hospital (NLH)**

Dinh Van Luong, Nguyen Binh Hoa, Ha Thi Tuyet Trinh, Doan Thu Ha, Dinh Thi Huong, Nguyen Trung Thanh

**FIT RD Social Enterprise Company Limited (FIT RD)**

Nguyen Thi Han, Nguyen Thi Cam Van, Ong Nguyen Huyen Trang, Tran Thi Thu Thuy

**Friends for International TB Relief (FIT)**

Luan Vo Nguyen Quang, Andrew James Codlin, Bui Thi Huyen

**Karolinska Institutet**

Lina Davies Forsman

**2. List of Ethics committees and Regulatory bodies**

**Vietnam**

Committee of Scientific and Ethical Review in Biomedical Research of Vietnam National Lung Hospital.

**Sweden**

Swedish Ethical Review Authority

**3. Full List of Inclusion and Exclusion Criteria**

Inclusion & exclusion criteria:

Participants above 18 years of age, able to provide informed consent, and have no current plans to relocate outside the designated area for the duration of the study will be included in the study.

Group 1 only:

+ People with biologically confirmed pulmonary TB disease (either drug-susceptible TB or drug-resistant TB) via Xpert MTB/RIF Ultra (Cepheid, Sunnyvale, California) who also have an abnormal chest X-ray (CXR) result*

*To reduce the false positive rate of Xpert Ultra assays

Group 2 only:

+ People without symptoms of active TB disease who are household contacts* of people with new microbiologically-confirmed pulmonary TB who initiated treatment in Ha Noi, Vietnam.

+ Have a normal CXR result.

* Household contacts of a person with TB are defined as members who live under the same roof as the person with contagious pulmonary TB or who meet the following conditions:

- Sleeping under the same roof or sharing a kitchen space as TB-affected persons at least one night/week for three months before the person was diagnosed with TB.

- Staying under the same roof with TB-affected persons for at least one hour/day and continuously five days/week for three months before the person was diagnosed with TB.

Group 3 only:

+ Known past negative IGRA test results among those at low risk for TB infection.

+ No known and/or reported history of contact or exposure to either TB disease or *M. tuberculosis* bacteria.

+ Have a normal CXR.

The main exclusion criteria were hypersensitive or contraindicated to IGRA. And for groups 2 and 3, the participant will be excluded if they are presumed to have TB disease with symptoms (cough, fever, night sweats, unintentional weight loss) and/or an abnormal CXR result suggestive of TB disease

+ Diagnosed with TB (microbiologically- or clinically-confirmed) in all forms or report having taken treatment for TB disease.

+ History of TB infection (self-recorded or documented)

**4. Supplementary methods**

We defined QFT-Plus result interpretation (Supplementary Table S1) as follows: a positive result required (TB1 or TB2)-nil ≥0.35 IU/mL and ≥25% of nil; a negative result required (TB1 and TB2)-nil <0.35 IU/mL or <25% of nil with mitogen-nil ≥0.5 IU/mL; an indeterminate result occurred if (TB1 and TB2)-nil <0.35 IU/mL or <25% of nil with mitogen-nil <0.5 IU/mL. or nil >8 IU/mL.

We defined TB-Feron FIA result interpretation (Supplementary Table S2) as follows: a positive result required TB Antigen-Nil ≥0.35 IU/mL and ≥25% of Nil; a negative result required TB Antigen-Nil <0.35 IU/mL or <25% of Nil with Mitogen-Nil ≥0.5 IU/mL; an indeterminate result occurred if TB Antigen-Nil <0.35 IU/mL or <25% of Nil with Mitogen-Nil <0.5 IU/mL, or Nil >8.0 IU/mL.

**Supplementary Table S1: QFT-Plus result interpretation guidelines**

| **Nil**  **(IU/ml)** | **TB1-Nil**  **(IU/ml)** | **TB2-Nil (IU/ml)** | **Mitogen- Nil**  **(IU/ml)** | **QFT-Plus Results** | **Interpretation** |
| --- | --- | --- | --- | --- | --- |
| ≤8.0 | ≥0.35 & ≥25% Nil | Any | Any | Positive | TB infection |
|  | Any | ≥0.35 & ≥25% Nil |  |  |  |
|  | <0.35 or ≥0.35 and < 25% Nil | <0.35 or ≥0.35 and < 25% Nil | ≥0.5 | Negative | No TB infection |
|  | <0.35 or ≥0.35 and < 25% Nil | <0.35 or ≥0.35 and < 25% Nil | <0.5 | Indeterminate | Indeterminate |
| >8.0 | Any | | |  |  |

**Supplementary Table S2: TB Feron result interpretation guidelines**

| **Nil**  **(IU/ml)** | **TB Antigen - Nil**  **(IU/ml)** | **Mitogen-Nil (IU/ml)** | **QFT-Plus Results** | **Interpretation** |
| --- | --- | --- | --- | --- |
| ≤8.0 | <0.35 | ≥0.5 | Negative | No TB infection |
|  | ≥0.35 & <25% of Nil value | ≥0.5 |  |  |
|  | ≥0.35 and ≥ 25% of Nil value | Any | Positive | TB infection |
|  | <0.35 | >0.5 | Indeterminate | Results are indeterminate for TB Antigen responsiveness |
|  | ≥0.35 and < 25% of Nil value | >0.5 |  |  |
| >8.0 | Any | Any |  |  |

*TB2-Nil or TBAg-Nil represents the interferon-gamma release by CD4 and CD8 T cells in both tests.*

**5. Supplementary Table S3a. Baseline characteristics of study participants (All populations by QFT-Plus)**

| **Characteristics** | **Total** | | **QFT-Plus** | | | | **p-value** |
| --- | --- | --- | --- | --- | --- | --- | --- |
|  |  |  | **Negative** | | **Positive** | |  |
|  | **n** | **%** | **n** | **%** | **n** | **%** |  |
| **Total** |  |  |  |  |  |  |  |
| **Sex** | 345 |  | 183 | 53.0% | 162 | 47.0% | 0.008 |
| Male | 157 | 45.5% | 71 | 38.8% | 86 | 53.1% |  |
| Female | 188 | 54.5% | 112 | 61.2% | 76 | 46.9% |  |
| **Age group (years)** |  |  |  |  |  |  | <0.0001 |
| 18 - 34 | 140 | 40.6% | 93 | 50.8% | 47 | 29.0% |  |
| 35 - 44 | 71 | 20.6% | 31 | 16.9% | 40 | 24.7% |  |
| 45 - 54 | 70 | 20.2% | 38 | 20.8% | 32 | 19.7% |  |
| 55 - 64 | 44 | 12.8% | 17 | 9.3% | 27 | 16.7% |  |
| >64 | 20 | 5.8% | 4 | 2.2% | 16 | 9.9% |  |
| *Median age (IQR)†* | 40 | 28-51 | 33 | 25-48 | 43 | 33-56 | <0.00001 |
| **Smoking*** | 75 | 21.7% | 22 | 12.0% | 53 | 32.7% | <0.00001 |
| **Alcohol** |  |  |  |  |  |  | 0.507 |
| No alcohol | 238 | 69.0% | 132 | 72.1% | 106 | 65.4% |  |
| 1-7_units/ week | 99 | 28.7% | 48 | 26.2% | 51 | 31.5% |  |
| 8-14_units/ week | 6 | 1.7% | 2 | 1.1% | 4 | 2.5% |  |
| > 14_units/ week | 2 | 0.6% | 1 | 0.6% | 1 | 0.6% |  |
| **Diabetes Mellitus type II** | 10 | 2.9% | 3 | 1.6% | 7 | 4.3% | 0.138 |
| **HBV/ HBC co-infection** | 10 | 2.9% | 5 | 2.7% | 5 | 3.1% | 0.845 |
| **BCG vaccinated** |  |  |  |  |  |  | 0.001 |
| No vaccination | 137 | 39.7% | 56 | 30.6% | 81 | 50.0% |  |
| Vaccinated | 127 | 36.8% | 77 | 42.1% | 50 | 30.9% |  |
| Unknown | 81 | 23.5% | 50 | 27.3% | 31 | 19.1% |  |
| **TB symptoms** |  | 0.0% |  | 0.0% |  | 0.0% |  |
| Cough |  | 0.0% |  | 0.0% |  | 0.0% |  |
| Hemoptysis |  | 0.0% |  | 0.0% |  | 0.0% |  |
| Night sweats |  | 0.0% |  | 0.0% |  | 0.0% |  |
| Fever | 3 | 0.9% | 1 | 0.6% | 2 | 1.2% | 0.492 |
| **TB score**^20^** |  |  |  |  |  |  |  |
| Mild |  | 0.0% |  | 0.0% |  | 0.0% |  |
| Moderate to severe |  | 0.0% |  | 0.0% |  | 0.0% |  |
| **Other infections** | 12 | 3.5% | 6 | 3.3% | 6 | 3.7% | 0.83 |

**6. Supplementary Table S3b. Baseline characteristics of study participants (by TB Feron results)**

| **Characteristics** | **TB Feron test results** | | | | | | | | | | | | | | | | | | | | | |
| --- | --- | --- | --- | --- | --- | --- | --- | --- | --- | --- | --- | --- | --- | --- | --- | --- | --- | --- | --- | --- | --- | --- |
|  | **Total** | | **Total population** | | | | **p-value** | **Group 1 (n=95)** | | | | **p-value** | **Group 2 (n=200)** | | | | **p-value** | **Group**  **3 (n=50)** | | | | **p-value** |
|  |  |  | **Negative** | | **Positive** | |  | **Negative** | | **Positive** | |  | **Negative** | | **Positive** | |  | **Negative** | | **Positive** | |  |
|  | **n** | **%** | **n** | **%** | **n** | **%** |  | **n** | **%** | **n** | **%** |  | **n** | **%** | **n** | **%** |  | **n** | **%** | **n** | **%** |  |
| **Total** |  |  |  |  |  |  |  |  |  |  |  |  |  |  |  |  |  |  |  |  |  |  |
| **Gender** | 345 |  | 149 | 43.19% | 196 | 56.81% | 0.003 | 11 | 11.58% | 84 | 88.42% | 0.744 | 103 | 51.50% | 97 | 48.50% | 0.073 | 35 | 70.00% | 15 | 30.00% | 0.529 |
| Male | 157 | 45.51% | 54 | 36.24% | 103 | 52.55% |  | 8 | 72.73% | 57 | 67.86% |  | 31 | 30.10% | 41 | 42.27% |  | 15 | 42.86% | 5 | 33.33% |  |
| Female | 188 | 54.49% | 95 | 63.76% | 93 | 47.45% |  | 3 | 27.27% | 27 | 32.14% |  | 72 | 69.90% | 56 | 57.73% |  | 20 | 57.14% | 10 | 66.67% |  |
| **Age group** |  |  |  |  |  |  | 0.036 |  |  |  |  | 0.564 |  |  |  |  | 0.138 |  |  |  |  |  |
| 18 - 34 | 140 | 40.58% | 70 | 46.98% | 70 | 35.71% |  | 6 | 54.55% | 29 | 34.52% |  | 29 | 28.16% | 26 | 26.80% |  | 35 | 100.00% | 15 | 100.00% |  |
| 35 - 44 | 71 | 20.58% | 24 | 16.11% | 47 | 23.98% |  | 1 | 9.09% | 14 | 16.67% |  | 23 | 22.33% | 33 | 34.02% |  |  | 0.00% |  |  |  |
| 45 - 54 | 70 | 20.29% | 35 | 23.49% | 35 | 17.86% |  | 1 | 9.09% | 17 | 20.24% |  | 34 | 33.01% | 18 | 18.56% |  |  | 0.00% |  |  |  |
| 55 - 64 | 44 | 12.75% | 14 | 9.40% | 30 | 15.31% |  | 1 | 9.09% | 15 | 17.86% |  | 13 | 12.62% | 15 | 15.46% |  |  | 0.00% |  |  |  |
| >64 | 20 | 5.80% | 6 | 4.03% | 14 | 7.14% |  | 2 | 18.18% | 9 | 10.71% |  | 4 | 3.88% | 5 | 5.15% |  |  | 0.00% |  |  |  |
| *Median age (IQR)†* | 38 | 26-49 | 40 | 30-52 | 40 | 28-51 | 0.053 | 34 | 30-59 | 44 | 30-57 | 0.569 | 44 | 32-52 | 40 | 33-51 | 0.4543 | 24 | 22-26 | 25 | 23-28 | 0.717 |
| **Smoking*** | 75 | 21.74% | 24 | 16.11% | 51 | 26.02% | 0.027 | 5 | 45.45% | 32 | 38.10% | 0.638 | 15 | 14.56% | 18 | 18.56% | 0.447 | 4 | 11.43% | 1 | 6.67% | 0.607 |
| **Alcohol** |  |  |  |  |  |  | 0.601 |  |  |  |  |  |  |  |  |  |  |  |  |  |  |  |
| Not drink | 238 | 68.99% | 105 | 70.47% | 133 | 67.86% |  | 7 | 63.64% | 51 | 60.71% | 0.201 | 73 | 70.87% | 69 | 71.13% | 0.118 | 25 | 71.43% | 13 | 86.67% | 0.477 |
| 1-7_units | 99 | 28.70% | 42 | 28.19% | 57 | 29.08% |  | 3 | 27.27% | 32 | 38.10% |  | 30 | 29.13% | 23 | 23.71% |  | 9 | 25.71% | 2 | 13.33% |  |
| 8-14_units | 6 | 1.74% | 1 | 0.67% | 5 | 2.55% |  | 1 | 9.09% | 1 | 1.19% |  | 0 | 0.00% | 4 | 4.12% |  | 0 | 0.00% | 0 | 0.00% |  |
| 14_units | 2 | 0.58% | 1 | 0.67% | 1 | 0.51% |  | 0 | 0.00% | 0 | 0.00% |  | 0 | 0.00% | 1 | 1.03% |  | 1 | 2.86% | 0 | 0.00% |  |
| **Diabetes Mellitus type II** | 10 | 2.90% | 2 | 1.34% | 8 | 4.08% | 0.133 | 0 | 0.00% | 8 | 9.52% | 0.285 | 2 | 1.94% | 0 | 0.00% | 0.168 | 0 | 0.00% | 0 | 0.00% | NA |
| **Hepatitis** | 10 | 2.90% | 3 | 2.01% | 7 | 3.57% | 0.393 | 0 | 0.00% | 2 | 2.38% | 0.605 | 3 | 2.91% | 5 | 5.15% | 0.419 | 0 | 0.00% | 0 | 0.00% | NA |
| **BCG vaccinated** |  |  |  |  |  |  | 0.642 |  |  |  |  | 0.843 |  |  |  |  | 0.373 |  |  |  |  | 0.734 |
| No vaccination | 137 | 39.71% | 55 | 36.91% | 82 | 41.84% |  | 5 | 45.45% | 46 | 54.76% |  | 45 | 43.69% | 35 | 36.08% |  | 5 | 14.29% | 1 | 6.67% |  |
| Vaccinated | 127 | 36.81% | 58 | 38.93% | 69 | 35.20% |  | 5 | 45.45% | 32 | 38.10% |  | 28 | 27.18% | 25 | 25.77% |  | 25 | 71.43% | 12 | 80.00% |  |
| Unknown | 81 | 23.48% | 36 | 24.16% | 45 | 22.96% |  | 1 | 9.09% | 6 | 7.14% |  | 30 | 29.13% | 37 | 38.14% |  | 5 | 14.29% | 2 | 13.33% |  |
| **TB symptoms** |  | 0.00% |  | 0.00% |  | 0.00% |  | 9 | 81.82% | 80 | 95.24% | 0.085 |  | 0.00% |  | 0.00% |  |  | 0.00% |  | 0.00% |  |
| Cough |  | 0.00% |  | 0.00% |  | 0.00% |  | 9 | 81.82% | 79 | 94.05% | 0.144 |  | 0.00% |  | 0.00% |  |  | 0.00% |  | 0.00% |  |
| Haemoptysis |  | 0.00% |  | 0.00% |  | 0.00% |  | 1 | 9.09% | 10 | 11.90% | 0.784 |  | 0.00% |  | 0.00% |  |  | 0.00% |  | 0.00% |  |
| Night sweats |  | 0.00% |  | 0.00% |  | 0.00% |  | 2 | 18.18% | 18 | 21.43% | 0.775 |  | 0.00% |  | 0.00% |  |  | 0.00% |  | 0.00% |  |
| Fever | 3 | 0.87% | 1 | 0.67% | 2 | 1.02% | 0.729 | 0 | 0.00% | 2 | 2.38% | 0.605 | 1 | 0.97% | 0 | 0.00% | 0.331 | 0 | 0.00% |  | 0.00% | NA |
| **TB score**** |  |  |  |  |  |  |  |  |  |  |  | 0.163 |  |  |  |  |  |  |  |  |  |  |
| Mild |  | 0.00% |  | 0.00% |  | 0.00% |  | 8 | 72.73% | 74 | 88.10% |  |  | 0.00% |  | 0.00% |  |  | 0.00% |  |  |  |
| Moderate to severe |  | 0.00% |  | 0.00% |  | 0.00% |  | 3 | 27.27% | 10 | 11.90% |  |  | 0.00% |  | 0.00% |  |  | 0.00% |  |  |  |
|  |  |  |  |  |  |  |  |  |  |  |  |  |  |  |  |  |  |  |  |  |  |  |
| **Other infections** | 12 | 3.48% | 2 | 1.09% | 10 | 5.10% | 0.059 | 0 | 0.00% | 0 | 0.00% | NA | 1 | 0.97% | 7 | 7.22% | 0.024 | 1 | 2.86% | 3 | 20.00% | 0.041 |
| **** Smoking:*** *including current and past smoking* | | | | | | | | | | | | | | | | | | | | | | |
| ***** TB score:*** *Bandim*  *TB Score, available at: Sarkar K, Kashyap B, Lnu S, Avasthi RK, Khanna A. Utility of a Clinical Scoring System (Bandim TB Score and Karnofsky Performance Score) to Assess Mycobacterial Burden in Terms of Cartridge-Based Nucleic Acid Amplification Test (CBNAAT) Cycle Threshold Values Among Pulmonary TB Patients. Cureus. 2023 Dec 22;15(12):e50976. doi: 10.7759/cureus.50976. PMID: 38259416; PMCID: PMC10801344.* | | | | | | | | | | | | | | | | | | | | | | |

**7. Supplementary Table S4. Cross-tabulation of TB-Feron and QFT-Plus test results (All populations)**

| **All populations (n=345)** | | **QFT-Plus** | | | **p-value** |
| --- | --- | --- | --- | --- | --- |
|  |  | **Positive (n, %)** | **Negative (n, %)** | **Total (n, %)** | <0.0001 |
| **TB-Feron** | **Positive (n, %)** | 146 (74.5%) | 50 (25.5%) | 196 (56.8%) |  |
|  | **Negative (n, %)** | 16 (10.7%) | 133 (89.3%) | 149 (43.2%) |  |
|  | **Total (n, %)** | 162 (47.0%) | 183 (53.0%) | 345 (100.0%) |  |

**8. Supplementary Table S5. TB Feron test results (including indeterminate results)**

| **TB Feron test results** | **QuantiFERON Gold Plus TB (QFT) test results** | | | | | | | | **p-value** |
| --- | --- | --- | --- | --- | --- | --- | --- | --- | --- |
|  | **Negative** | | **Positive** | | **Indeterminate** | | **Total** | |  |
|  | **n** | **%** | **n** | **%** |  |  | **n** | **%** |  |
| **All populations (n=350)** | 185 | 52.9% | 163 | 46.6% | 2 | 0.5% | 350 | 100.0% |  |
| Negative | 133 | 88.7% | 16 | 10.7% | 1 | 0.6% | 150 | 100.0% | <0.0001 |
| Positive | 50 | 25.4% | 146 | 74.1% | 1 | 0.5% | 197 | 100.0% |  |
| Indeterminate | 2 | 66.7% | 1 | 33.3% | 0 | 0.0% | 3 | 100.0% |  |
| **Group 1 (n=96)** | 7 | 7.3% | 89 | 92.7% | 0 | 0.0% | 96 | 100.0% |  |
| Negative | 3 | 27.27% | 8 | 72.73% | 0 | 0.0% | 11 | 100.0% | 0.025 |
| Positive | 4 | 4.76% | 80 | 95.24% | 0 | 0.0% | 84 | 100.0% |  |
| Indeterminate | 0 | 0.00% | 1 | 100.00% | 0 | 0.0% | 1 | 100.0% |  |
| **Group 2 (n=204)** | 128 | 62.7% | 74 | 36.3% | 2 | 1.0% | 204 | 100.0% |  |
| Negative | 95 | 91.3% | 8 | 7.7% | 1 | 1.0% | 104 | 100.0% | <0.0001 |
| Positive | 31 | 31.6% | 66 | 67.4% | 1 | 1.0% | 98 | 100.0% |  |
| Indeterminate | 2 | 100.0% | 0 | 0.0% | 0 | 0.0% | 2 | 100.0% |  |
| **Group 3 (n=50)** | 50 | 100.0% | 0 | 0.0% | 0 | 0.0% | 50 | 100.0% |  |
| Negative | 35 | 100.0% | 0 | 0.0% | 0 | 0.0% | 35 | 100.0% | NA |
| Positive | 15 | 100.0% | 0 | 0.0% | 0 | 0.0% | 15 | 100.0% |  |
| Indeterminate | 0 | NA | 0 | NA | 0 | NA | 0 | 100.0% |  |

*QFT had two indeterminate results: one was TB Feron positive and one was TB Feron negative. TB Feron had three indeterminate results: two were QFT positive and one was QFT negative. No indeterminate results were shared between the two tests.*

**9. Supplementary Table S6a. Distribution of discordant IGRA results by study group and selected clinical factors**

| **Factors** | **Group 1 (n=11)** | **Group 2 (n=7)** | **Group 3 (n=3)** | **Total**  **(n=21)** |
| --- | --- | --- | --- | --- |
| **Cough** | 10 | 0 | 0 | 10 |
| **TB score (moderate–severe)** | 1 | 0 | 0 | 1 |
| **HBV/HCV coinfection** | 0 | 4 | 0 | 4 |
| **Other infections** | 0 | 3 | 3 | 6 |

**10. Supplementary Table S6b. Individual-level listing of discordant IGRA results with corresponding clinical factors**

| **Subject ID** | **Study group** | **Discordance pattern (QFT+/Feron- or QFT-/Feron+)** | **Cough** | **TB score moderate–severe** | **HBV/HCV coinfection** | **Other infections** |
| --- | --- | --- | --- | --- | --- | --- |
| E.1-1-001 | Group 1 | QFT+/Feron- | Yes | Yes | No | No |
| E.1-1-004 | Group 1 | QFT+/Feron- | Yes | No | No | No |
| E.1-1-010 | Group 1 | QFT+/Feron- | Yes | Yes | No | No |
| E.1-1-030 | Group 1 | QFT-/Feron+ | Yes | No | No | No |
| E.1-1-031 | Group 1 | QFT+/Feron- | Yes | Yes | No | No |
| E.1-1-038 | Group 1 | QFT+/Feron- | Yes | No | No | No |
| E.1-1-042 | Group 1 | QFT-/Feron+ | Yes | No | No | No |
| E.1-1-043 | Group 1 | QFT-/Feron+ | Yes | Yes | No | No |
| E.1-1-050 | Group 1 | QFT+/Feron- | No | No | No | No |
| E.1-1-054 | Group 1 | QFT+/Feron- | No | No | No | No |
| E.1-1-080 | Group 1 | QFT-/Feron+ | Yes | No | No | No |
| E.1-1-094 | Group 1 | QFT+/Feron- | Yes | No | No | No |
| E.1-2-010 | Group 2 | QFT-/Feron+ | No | No | No | No |
| E.1-2-013 | Group 2 | QFT-/Feron+ | No | No | No | No |
| E.1-2-018 | Group 2 | QFT-/Feron+ | No | No | No | No |
| E.1-2-019 | Group 2 | QFT-/Feron+ | No | No | Yes | No |
| E.1-2-024 | Group 2 | QFT-/Feron+ | No | No | No | No |
| E.1-2-033 | Group 2 | QFT-/Feron+ | No | No | No | No |
| E.1-2-034 | Group 2 | QFT-/Feron+ | No | No | No | No |
| E.1-2-035 | Group 2 | QFT-/Feron+ | No | No | No | No |
| E.1-2-036 | Group 2 | QFT-/Feron+ | No | No | Yes | No |
| E.1-2-040 | Group 2 | QFT-/Feron+ | No | No | No | No |
| E.1-2-045 | Group 2 | QFT-/Feron+ | No | No | No | No |
| E.1-2-073 | Group 2 | QFT-/Feron+ | No | No | No | Yes |
| E.1-2-076 | Group 2 | QFT-/Feron+ | No | No | No | No |
| E.1-2-079 | Group 2 | QFT-/Feron+ | No | No | No | No |
| E.1-2-080 | Group 2 | QFT-/Feron+ | No | No | No | No |
| E.1-2-083 | Group 2 | QFT-/Feron+ | No | No | No | No |
| E.1-2-086 | Group 2 | QFT-/Feron+ | No | No | No | No |
| E.1-2-094 | Group 2 | QFT-/Feron+ | No | No | No | Yes |
| E.1-2-098 | Group 2 | QFT-/Feron+ | No | No | No | No |
| E.1-2-102 | Group 2 | QFT-/Feron+ | No | No | No | No |
| E.1-2-103 | Group 2 | QFT+/Feron- | No | No | No | Yes |
| E.1-2-107 | Group 2 | QFT-/Feron+ | No | No | No | No |
| E.1-2-108 | Group 2 | QFT-/Feron+ | No | No | No | No |
| E.1-2-109 | Group 2 | QFT-/Feron+ | No | No | No | No |
| E.1-2-117 | Group 2 | QFT-/Feron+ | No | No | No | No |
| E.1-2-120 | Group 2 | QFT-/Feron+ | No | No | No | No |
| E.1-2-125 | Group 2 | QFT-/Feron+ | No | No | No | No |
| E.1-2-126 | Group 2 | QFT-/Feron+ | No | No | Yes | No |
| E.1-2-136 | Group 2 | QFT+/Feron- | No | No | No | No |
| E.1-2-137 | Group 2 | QFT+/Feron- | No | No | Yes | No |
| E.1-2-143 | Group 2 | QFT-/Feron+ | No | No | No | No |
| E.1-2-145 | Group 2 | QFT-/Feron+ | No | No | No | No |
| E.1-2-150 | Group 2 | QFT+/Feron- | No | No | No | No |
| E.1-2-163 | Group 2 | QFT+/Feron- | No | No | No | No |
| E.1-2-169 | Group 2 | QFT+/Feron- | No | No | No | No |
| E.1-2-172 | Group 2 | QFT-/Feron+ | No | No | No | No |
| E.1-2-174 | Group 2 | QFT+/Feron- | No | No | No | No |
| E.1-2-185 | Group 2 | QFT-/Feron+ | No | No | No | No |
| E.1-2-202 | Group 2 | QFT+/Feron- | No | No | No | No |
| E.1-3-002 | Group 3 | QFT-/Feron+ | No | No | No | No |
| E.1-3-006 | Group 3 | QFT-/Feron+ | No | No | No | No |
| E.1-3-007 | Group 3 | QFT-/Feron+ | No | No | No | No |
| E.1-3-010 | Group 3 | QFT-/Feron+ | No | No | No | No |
| E.1-3-011 | Group 3 | QFT-/Feron+ | No | No | No | No |
| E.1-3-012 | Group 3 | QFT-/Feron+ | No | No | No | Yes |
| E.1-3-017 | Group 3 | QFT-/Feron+ | No | No | No | No |
| E.1-3-018 | Group 3 | QFT-/Feron+ | No | No | No | No |
| E.1-3-022 | Group 3 | QFT-/Feron+ | No | No | No | No |
| E.1-3-023 | Group 3 | QFT-/Feron+ | No | No | No | Yes |
| E.1-3-025 | Group 3 | QFT-/Feron+ | No | No | No | No |
| E.1-3-029 | Group 3 | QFT-/Feron+ | No | No | No | No |
| E.1-3-030 | Group 3 | QFT-/Feron+ | No | No | No | Yes |
| E.1-3-034 | Group 3 | QFT-/Feron+ | No | No | No | No |
| E.1-3-035 | Group 3 | QFT-/Feron+ | No | No | No | No |

**11. Table S7a. Relationship between IFN-γ level (TB2) and TB Feron results (All populations)**

| **Indicator** | **Categories** | **Mean**  **(IU/ml)** | **95% CI** | **Empirical estimation** | **Theoretical sensitivity at threshold** | **Theoretical specificity at threshold** | **p value^*^** |
| --- | --- | --- | --- | --- | --- | --- | --- |
| **Corrected (TB2-Nil)** | All | 2.14 | 1.80; 2.49 |  |  |  |  |
|  | False positive | -0.07 | -0.17;0.04 |  |  |  |  |
|  | False negative | 2 | 0.49;3.51 |  |  |  |  |
|  | TB Feron (+) | 3.56 | 3.05;4.06 | 0.36 | 0.74 | 0.91 | 0.3395 |
|  | TB Feron (-) | 0.28 | 0.06;0.50 |  |  |  |  |
|  | QFT-Plus(+) | 4.52 | 4.00;5.04 | 0.34 | 0.99 | 0.99 |  |
|  | QFT-Plus (-) | 0.037 | -0.08;0.15 |  |  |  | 0.5008 |
| **Uncorrected (TB2)** | All | 2.57 | 2.20;2.93 |  |  |  |  |
|  | False positive | 0.45 | 0.16;0.75 |  |  |  |  |
|  | False negative | 2.3 | 0.78;3.80 |  |  |  | 0.476 |
|  | TB Feron (+) | 4.15 | 3.63;4.68 | 0.63 | 0.76 | 0.89 |  |
|  | TB Feron (-) | 0.48 | 0.26;0.70 |  |  |  | 0.5084 |
|  | QFT-Plus (+) | 5.11 | 4.56;5.66 | 0.53 | 0.99 | 0.95 |  |
|  | QFT-Plus (-) | 0.31 | 0.18;0.45 |  |  |  |  |
| **Nil** | All | 0.42 | 0.34;0.50 |  |  |  |  |
|  | False positive | 0.52 | 0.20;0.84 |  |  |  | 0.5702 |
|  | False negative | 0.29 | 0.16;0.42 |  |  |  | 0.5416 |
|  | TB Feron (+) | 0.6 | 0.46;0.73 | 0.27 | 0.53 | 0.83 |  |
|  | TB Feron (-) | 0.2 | 0.17;0.23 |  |  |  |  |
|  | QFT-Plus (+) | 0.6 | 0.46;0.72 | 0.27 | 0.55 | 0.78 |  |
|  | QFT-Plus (-) | 0.28 | 0.19;0.37 |  |  |  |  |

**12. Table S7b. Relationship between IFN-γ level (TB2) and TB Feron results (Group 2- Household contacts)**

| **Indicator** | **Categories** | **Mean**  **(IU/ml)** | **95% CI** | **Empirical estimation** | **Theoretical sensitivity at threshold** | **Theoretical specificity at threshold** | p value* |
| --- | --- | --- | --- | --- | --- | --- | --- |
| **Corrected (TB2-Nil)** | All | 1.3 | 0.95;1.64 |  |  |  |  |
|  | False positive | 0.005 | -0.06;0.07 |  |  |  |  |
|  | False negative | 0.56 | 0.37;0.75 |  |  |  |  |
|  | TB Feron (+) | 2.5 | 1.90;3.10 | 0.27 | 0.69 | 0.91 | 0.3448 |
|  | TB Feron (-) | 0.17 | -0.026;0.36 |  |  |  |  |
|  | QFT-Plus(+) | 3.33 | 2.65;4.01 | 0.31 | 0.97 | 0.99 | 0.5281 |
|  | QFT-Plus (-) | 0.1 | -0.05;0.25 |  |  |  |  |
| **Uncorrected (TB2)** | All | 1.66 | 1.28;2.03 |  |  |  |  |
|  | False positive | 0.33 | 0.27;0.38 |  |  |  |  |
|  | False negative | 0.93 | 0.68;1.19 |  |  |  |  |
|  | TB Feron (+) | 3.03 | 2.38;3.68 | 0.33 | 0.82 | 0.79 | 0.4362 |
|  | TB Feron (-) | 0.36 | 0.16;0.56 |  |  |  | 0.508 |
|  | QFT-Plus (+) | 3.93 | 3.91;4.68 | 0.5 | 0.99 | 0.94 |  |
|  | QFT-Plus (-) | 0.32 | 0.16;0.47 |  |  |  |  |
| **Nil** | All | 0.36 | 0.28;0.43 |  |  |  |  |
|  | False positive | 0.32 | 0.24;0.41 |  |  |  |  |
|  | False negative | 0.38 | 0.13;0.62 |  |  |  |  |
|  | TB Feron (+) | 0.53 | 0.39;0.68 | 0.21 | 0.66 | 0.72 | 0.7 |
|  | TB Feron (-) | 0.19 | 0.16;0.22 |  |  |  | 0.5341 |
|  | QFT-Plus (+) | 0.6 | 0.42;0.79 | 0.27 | 0.61 | 0.79 |  |
|  | QFT-Plus (-) | 0.21 | 0.18;0.24 |  |  |  |  |

*^*^ Student T-test*

**13. Supplementary Table S8: Intra-Test Reproducibility of TB Feron**

| **Differences*** | |
| --- | --- |
| **Sample size** | 15 |
| **Arithmetic mean** | 2.1 |
| **95% CI** | -1.2841 to 5.4441 |
| **P (H_0_: Mean=0)** | 0.206 |
| **Lower limit** | -9.8266 |
| **95% CI** | -15.7094 to -3.9437 |
| **Upper limit** | 13.9866 |
| **95% CI** | 8.1037 to 19.8694 |
| **Coefficient of Repeatability** | 12.2039 |
| **95% CI** | 9.0151 to 18.8879 |

******Bland-Altman^18^*

*Abbreviation: CI= confidence intervals;*

**14. Supplementary** **Figure 1. Intra-Test Reproducibility of TB Feron (Bland Altman plot)**

Legend: Bland-Altman analysis of IFN-γ levels showed a mean difference of 2.1 IU/mL, indicating no significant systematic bias between paired measurements. The 95% limits of agreement ranged from -9.8 IU/mL to 14.0 IU/mL.

IFN_1 refers to the interferon-gamma concentration measured by the TB-Feron assay during the first test conducted by one laboratory technician, while IFN_2 refers to the concentration obtained from the second test conducted independently by a different technician.

Alt text: Bland-Altman plot depicting intra-test reproducibility of TB-Feron with limits of agreement for IFN-γ measurements.
