# Supplementary figures and images for: Diagnostic Accuracy of the STANDARD F TB-Feron FIA Assay for Tuberculosis Infection in Vietnam: A Cross-Sectional Study
Source: Clin Infect Dis. 2025 Nov 26;82(5):e996–e1004. doi: 10.1093/cid/ciaf561 (PMC13189665; doi:10.1093/cid/ciaf561)

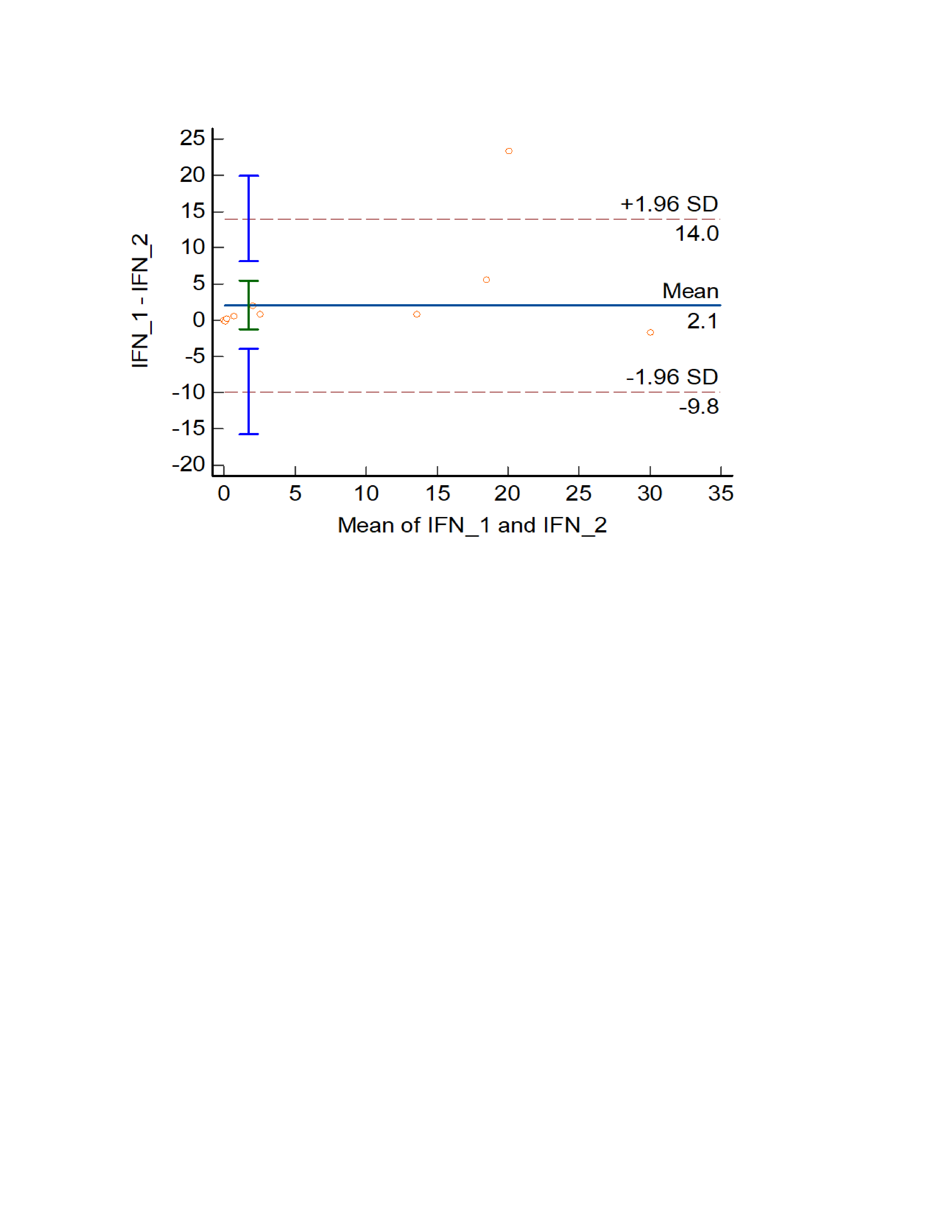

Supplement: ciaf561_Supplementary_Data [file ciaf561_supplementary_data.zip › TB Feron- Supplementary Figure 1.tiff]
